# Performance of ELISAs for detection of antibodies against porcine respiratory and reproductive syndrome virus in serum of pigs after PRRSV type 2 live vaccination and challenge

**DOI:** 10.1186/s40813-015-0015-9

**Published:** 2015-12-11

**Authors:** Tatjana Sattler, Jutta Pikalo, Eveline Wodak, Friedrich Schmoll

**Affiliations:** 1 Large Animal Clinic for Internal Medicine, University of Leipzig, An den Tierkliniken 11, 04103 Leipzig, Germany; 2 Institute for Veterinary Disease Control, AGES, Robert-Koch-Gasse 17, 2340 Mödling, Austria

**Keywords:** Swine, Highly pathogenic, Sensitivity, Specificity, Agreement

## Abstract

**Background:**

The aim of the study was to evaluate the performance of different newly developed and/or commercially available ELISAs for detection of PRRSV specific antibodies. Consequently, ten PRRSV negative piglets (group V) were vaccinated with a PRRSV type 2 vaccine. Blood samples were taken before as well as seven, 21 and 42 days after vaccination. At day 42 after vaccination (day 0 of the study) all of the piglets from group V and 10 non-prevaccinated PRRSV negative piglets (group N) were challenged with an HP PRRSV type 2 field strain. Blood samples were taken before and at days 3, 7, 10, 14, 21 and 28 after challenge. The success of vaccination and challenge was measured with RT qPCR. All serum samples were tested with six ELISAs for detection of PRRSV antibodies. Three of them are nucleocapsid-based, two use a glycoprotein extract and one uses inactivated whole virus as antigen. The specificity of the ELISAs was evaluated using 301 serum samples of piglets from PRRSV negative herds.

**Results:**

The piglets from group V tested positive by RT qPCR at day 7 after vaccination and all piglets tested positive at day 3 after challenge. PRRSV specific antibodies were seen with all nucleocapsid-based ELISAs from day 21 after vaccination onwards in group V and from day 10 after challenge in group N. The glycoprotein-based ELISAs detected antibodies from day 42 after vaccination (group V) and day 21 after challenge (group N). The agreement according to kappa-coefficient was almost perfect. The glycoprotein-based ELISAs were able to distinguish PRRSV type 2, although with some cross reactions. Regarding specificity, the ELISAs performed differently (specificity between 97.4 % and 100 %), whereas most of the ELISAs with higher sensitivity had a slightly lower specificity.

**Conclusions:**

All tested ELISA were able to detect PRRSV antibodies in the serum of pigs vaccinated with a PRRSV type 2 vaccine and after challenge with an HP PRRSV type 2 field strain. The onset on antibody detection differed, depending on the type of antigen used in the ELISAs. Most of the ELISAs with a higher sensitivity had a lower specificity.

## Background

Detection of antibodies (Ab) against porcine reproductive and respiratory syndrome virus (PRRSV) is, in addition to a number of different established PCR methods [1, 2], one important tool for the monitoring and surveillance of PRRSV in pig farms [3, 4]. In addition to the cost-effective, simple and rapid analysis by ELISA, alternative methods, such as serum neutralization test, immunofluorescence assay or Western blot are used for special indications [3, 5–7]. In recent years, several ELISAs for detection of Ab against PRRSV in pig serum have been developed [7–9], some of them with the intention of making them commercially available. Some ELISAs, however, have been on the market for many years and have been continuously adapted and improved. Studies have been published validating and comparing some of them [10–12]. The IDEXX PRRS X3 Ab test (IDEXX, Westbrook, USA) is usually used as the gold standard for comparison [8, 9, 13]. According to the manufacturer, this ELISA has a specificity of 99.9 % and a sensitivity of 98.8 %. Most of the ELISAs are able to detect Ab against PRRSV type 1 and type 2 [14]. However, some have been described as able to distinguish between PRRSV types [5, 7, 13]. The ELISAs presently used in routine analysis are usually based on the PRRSV nucleocapsid protein as antigen [15]. For some indications, ELISAs based on the non-structural proteins (Nsp) 7 or 9, the membrane glycoprotein 5 (Gp5) and recombinant antigens have been designed [8, 9, 16–18]. Most of them are not commercially available.

Some studies are available that give data about the onset of antibody development after vaccination with inactivated PRRSV vaccine or live attenuated vaccine as well as after challenge, measured by different methods [6, 8, 13]. At this point, no data are available regarding how newly developed ELISAs that already are or will in the near future become commercially available, perform after vaccination with a live attenuated PRRSV type 2 vaccine and the challenge of pigs with highly pathogenic (HP) PRRSV. Furthermore, the scientific community lacks data about the onset of Ab detection after infection with HP PRRSV while using some of the ELISAs that have been commercially available for many years.

The objective of the study was to test the performance of different commercial and newly developed ELISAs for the detection of Ab against PRRSV in the serum of pigs vaccinated with a newly developed PRRSV type 2 attenuated live vaccine, and/or challenged with an HP PRRSV field strain. Serum samples of PRRSV negative pigs were analysed to evaluate the specificity of the ELISAs.

## Results

### Molecular analysis

At the beginning of the study, all of the serum samples of the piglets from groups V and N tested negative in PRRSV RT qPCR. On days 7 (first sampling time) and 21 after vaccination (days −35 and −21 of the study), all of the piglets of group V tested positive in PRRSV RT qPCR. All of the piglets from both groups developed a PRRS viremia, detected from day 3 after the challenge with HP PRRSV onwards.

### Detection of PRRSV Ab by ELISA

**Fig. 1:**
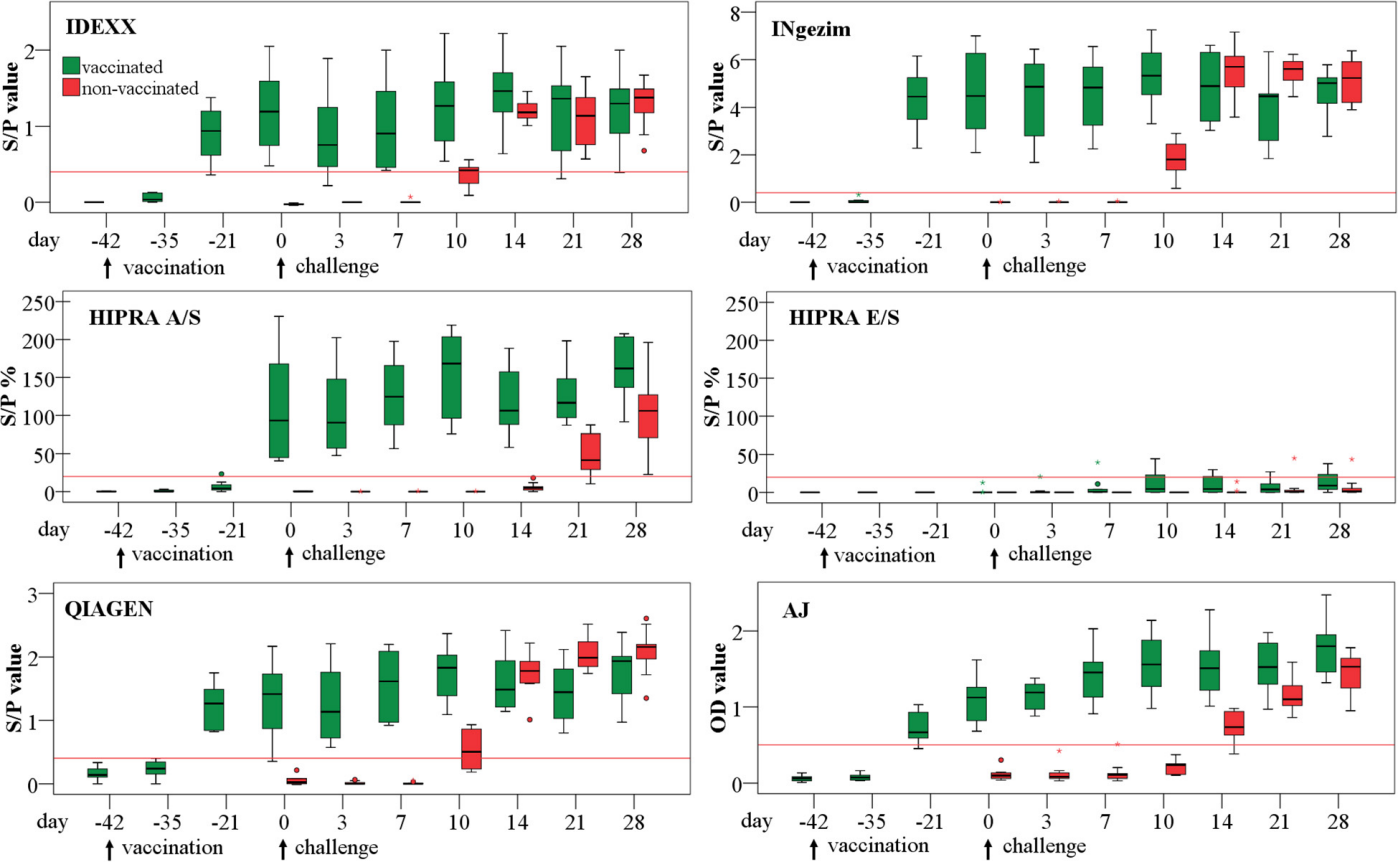
Boxplots of S/P values, respectively OD values of all PRRSV Ab ELISAs tested. Vaccinated group: vaccination with a PRRSV live vaccine at day −42 (see arrow), both groups: challenge with an HP PRRSV field strain at day 0 (see arrows), red lines: cut-off of the ELISAs

Serum samples of all piglets from groups V and N were PRRSV Ab negative in all of the tested ELISAs on day −42 (the start of the study). Figure 1 gives an overview of the S/P or OD values of each ELISA during the whole study. The number of seropositive samples on each sampling day tested with all ELISAs is given in Table 1. On day −21 of the study (21 days after vaccination), a seroconversion was observed in all piglets from group V, measured with the INgezim and the QIAGEN ELISA. One sample tested with the IDEXX ELISA was still PRRSV Ab negative at this point. The same sample, as well as another one, also tested negative with the AJ ELISA. These negative samples were just below the cut-off of the ELISAs. The first seroconversion in group N was observed on day 10 after challenge. The S/P value of all positive samples measured with the INgezim were significantly (*P* < 0.01) higher than in the other ELISAs, although the cut-off with the INgezim is the same as with IDEXX and QIAGEN.

**Table 1:** Results of PRRSV Ab ELISAs at the sampling points, number of positive animals

Study day	−42	−35	−21	0	3	7	10	14	21	28
Group V	*n* = 10	*n* = 10	*n* = 10	*n* = 10	*n* = 10	*n* = 10	*n* = 10	*n* = 10	*n* = 10	*n* = 10
IDEXX	0	0	9	10	9	10	10	10	9	9
INgezim	0	0	10	10	10	10	10	10	10	10
HIPRA A/S	0	0	1	10	10	10	10	10	10	10
HIPRA E/S	0	0	0	0	1	1	3	3	1	3
QIAGEN	0	0	10	10	10	10	10	10	10	10
AJ	0	0	8	10	10	10	10	10	10	10
Group N	*n* = 10	*n* = 10	*n* = 10	*n* = 10	*n* = 10	*n* = 10	*n* = 10	*n* = 10	*n* = 10	*n* = 10
IDEXX	n.d.	n.d.	n.d.	0	0	0	6	10	10	10
INgezim	n.d.	n.d.	n.d.	0	0	0	10	10	10	10
HIPRA A/S	n.d.	n.d.	n.d.	0	0	0	0	0	8	10
HIPRA E/S	n.d.	n.d.	n.d.	0	0	0	0	0	1	1
QIAGEN	n.d.	n.d.	n.d.	0	0	0	7	10	10	10
AJ	n.d.	n.d.	n.d.	0	0	0	0	8	10	10

Group V: vaccination with a PRRSV live vaccine at day −42

Groups V and N: challenge with an HP PRRSV field strain at day 0

n.d.: not done

A later onset of Ab detection was observed with the HIPRA A/S compared to the other ELISAs. On day −21, only one sample from group V was weakly positive. On day 0, however, all of the piglets from group V were seropositive and remained so until the end of the study. The same phenomenon was seen in group N with seroconversion on day 21 after challenge, measured with the HIPRA A/S. Some positive results were found with the HIPRA E/S throughout the study, especially in cases with high S/P values detected by the HIPRA A/S.

**Fig. 2:**
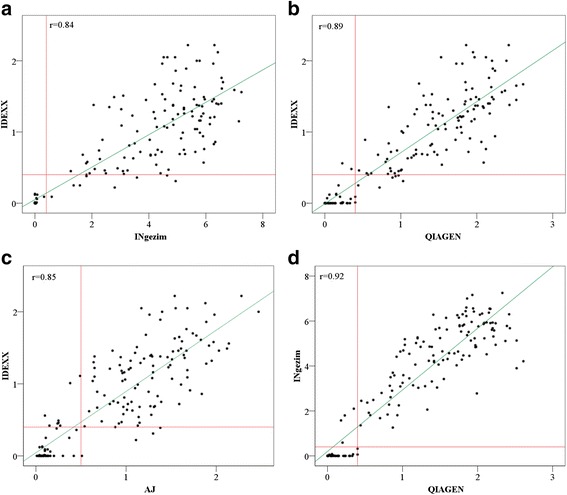
Scatterplot indicating the correlation between S/P values of study ELISAs. **a** IDEXX and INgezim ELISA, (**b**) IDEXX and QIAGEN ELISA, (**c**) IDEXX and AJ ELISA, (**d**) INgezim and QIAGEN ELISA. Green line: line of best fit; red lines: cut-off of the ELISAs

Several correlations were observed between the S/P or OD values of all study ELISAs (Table 2). Figure 2 shows the correlation between the IDEXX and INgezim, QIAGEN and AJ ELISA results over all sampling points and the correlation between INgezim and QIAGEN ELISA which was especially high. Both HIPRA ELISAs correlated on several time points with correlation coefficients up to 0.64. The agreement between the ELISAs, measured with kappa coefficient (κ), and the correlation of the positive/negative results can be seen in Table 3. An almost perfect agreement was found between IDEXX, INgezim, QIAGEN and AJ ELISAs. The agreement between HIPRA A/S and AJ ELISA was almost perfect as well. The HIPRA A/S agreed substantially (κ between 0.6 and 0.8) or less than substantially with the other ELISAs (not shown in the table). The agreement (κ) of the HIPRA E/S with the other ELISAs was mostly less than 0.2.

**Table 2:** Correlations between the S/P or OD values of the PRRSV Ab ELISAs tested using the correlation coefficient after Spearman

	INgezim	HIPRA A/S	HIPRA E/S	QIAGEN	AJ
IDEXX	0.84	0.73	0.71	0.89	0.85
INgezim		0.75	0.65	0.92	0.79
HIPRA A/S			0.62	0.73	0.87
HIPRA E/S				0.73	0.75
QIAGEN					0.84

**Table 3:** Agreement and correlation between the results of the PRRSV Ab ELISAs tested

		IDEXX			Agreement (κ)	Correlation coefficient
		Positive	Negative	Sum		
INgezim	Positive	112	8	120	0.89	0.90
	Negative	0	50	50		
QIAGEN	Positive	110	6	116	0.89	0.90
	Negative	2	52	54		
AJ	Positive	103	3	106	0.85	0.85
	Negative	9	55	64		
Sum		112	58	170		
			AJ			
HIPRA	Positive	89	0	89	0.80	0.81
	Negative	17	64	81		
QIAGEN	Positive	105	11	116	0.85	0.85
	Negative	1	53	54		
Sum		106	64	170		
			INgezim			
QIAGEN	Positive	116	0	116	0.95	0.95
	Negative	4	50	54		
AJ	Positive	106	0	106	0.82	0.83
	Negative	14	50	64		
Sum		120	50	170		

Results of groups V and N included, Correlation coefficient after Spearman given

Table 4 shows the results of the PRRSV negative samples and the calculated specificity of the ELISAs. The false-positive results of the QIAGEN ELISA had S/P values between 0.40 and 0.55; most were only slightly above the cut-off. The false-positive samples measured with the HIPRA A/S were between 22 % and 37 % of the positive control. No false-positive results were observed with the AJ.

**Table 4:** Specificity of three of the tested study ELISAs for detection of PRRSV antibodies

	Positive	Negative	Total	Specificity
QIAGEN	6	304	310	98.1 %
AJ	0	278	278	100 %
HIPRA A/S	5	330	335	98.5 %

Results of group S, group V at day −42 and group N at day 0 included

## Discussion

The performance of several newly developed and commercially available ELISAs for detection of PRRSV Ab was tested in this study using well-defined serum samples of PRRSV type 2 vaccinated, HP PRRSV type 2 challenged and negative pigs.

ELISAs that were coated with a similar antigen performed identically. With the nucleocapsid-based ELISAs IDEXX, INgezim and QIAGEN, a seroconversion was detected beginning on day 10 after the challenge with a HP PRRSV type 2 field strain. Antibodies against the nucleocapsid can be detected by the end of the second week after infection, but are not neutralizing [14]. No studies have been published regarding the newly developed INgezim and QIAGEN until now. Both ELISAs showed a higher sensitivity than the IDEXX by detecting all serum samples as positive at day 21 after vaccination and from day 10 after challenge onwards. In a recently published study, the sensitivity of the IDEXX ELISA was stated at 80 % in the field samples tested, compared to the immunoperoxidase monolayer assay (IPMA) [15]. This is in contrast to other studies which stated a sensitivity of the IDEXX ELISA of 100 % in serum samples 21 days after PRRSV inoculation [5] and of 91.5 % in field samples [7].

The specificity of the QIAGEN ELISA was, at 98.1 %, lower than the IDEXX which had a 100 % specificity as has been published previously [5, 7, 12], respectively a 99.9 % specificity, according to the manufacturer. The specificity of the INgezim ELISA was with 99.0 % between these two ELISAs [12]. Compared to the specificity of 92.3 % of a former version of this ELISA (INgezim PRRS Universal kit, Ingenasa), the INgezim ELISA used in our study has been improved considerably [11]. According to another study, specificities of 83 % and 77 % were found in field samples for the IDEXX ELISA and the INgezim PRRS Universal, respectively, compared to the IPMA [15]. The agreement between the IDEXX, INgezim and QIAGEN ELISAs was almost perfect, according to κ [19].

The AJ ELISA which is based on an inactivated virus performed almost similarly. A later onset of antibody detection with eight out of ten positive samples at day 21 after vaccination in group V and at day 14 after challenge in group N was seen, possibly depending on the fact that this first version of the assay allowed IgG antibody response detection only. After seroconversion, all samples remained positive until the end of the study, testifying a high sensitivity of this ELISA.

The antigens used in the HIPRA ELISAs contain, a glycoprotein rich extraction of the virus, according to the manufacturer. The major envelope proteins of the PRRSV are the GP5 and the M protein [20]. A conformational epitope of GP5 induces the production of neutralizing Ab that can be detected at the earliest four weeks after infection [14, 21]. This could be one reason for the late onset of Ab detection in the HIPRA ELISAs as was assumed to be the case in another study also conducted with this ELISA [17]. On the other hand, the duration of Ab against glycoproteins detectable by ELISA is proven to be much longer than in nucleocapsid based ELISAs [22]. When tested with the HIPRA ELISA, 100 % of the pigs were still Ab positive up to 100 days post vaccination [17]. We were not able to verify this in our study, since Ab were only tested for until 28 days after challenge or 42 days after vaccination, respectively. Differences between the ELISAs regarding sensitivity and specificity in field samples have been explained by the dissimilar characteristics of the antigens used [18]. The slightly delayed onset of PRRSV Ab detection measured by the AJ ELISA is probably caused by this fact as well. The specificity of the AJ ELISA was at 100 % higher than that of the HIPRA A/S at 98.5 % and similar to the IDEXX ELISA.

The agreement of the HIPRA E/S results with the other ELISAs is low because of its exclusive detection of PRRSV type 1 Ab that were not developed in the study pigs. Despite this exclusive detection of Ab against PRRSV type 1, some positive samples were found with the HIPRA E/S in this study after vaccination and/or challenge with PRRSV type 2. Cross-reactions between both types could explain these reactions. According to the manufacturer, a distinction between both genotypes can be made, if the S/P value of the ELISA in question (HIPRA A/S or E/S) is less than 30 % of the other. In our study, S/P values of 12 of the 14 samples found to be positive with the HIPRA E/S were less than 30 % of the S/P values of the HIPRA A/S. Therefore, these samples could be declared as PRRSV type 2 Ab positive. The other two positive samples were at 32 % and therefore only slightly above the limit calculated by the manufacturer. Some other unspecific cross reactions are possible.

## Conclusions

As a conclusion it can be said that all of the ELISAs tested were able to detect PRRSV specific Ab after vaccination with a PRRSV type 2 vaccine and/or challenge with an HP PRRSV type 2 field strain, although with differing levels of sensitivity and specificity. The onset of Ab detection by the ELISAs differed, depending on the antigen component used. This must be considered when choosing the ELISA. IDEXX and AJ ELISAs distinguish themselves with a particularly high specificity, the INgezim and QIAGEN ELISAs stand out with a high sensitivity. The HIPRA ELISAs were able to distinguish PRRSV type 2 related antibodies, although with some limitations caused by cross reactions.

## Methods

### Study design, animals and serum samples

Twenty piglets from a PRRSV free farm were included in the study. Ten of those piglets (group V) were injected with 2 ml of an attenuated PRRSV type 2 vaccine with the commercial name “Kyoto Biken” PIGWIN PRRSV2 (Kyoto Biken, Kyoto, Japan) at the age of three weeks. The highest ORF5 sequence identity of the Kyoto Biken vaccine to published sequences was observed for the Kyoto 93 strain (GenBank: AB175724). The ten piglets of group N remained unvaccinated. At the age of ten weeks, all 20 study piglets received an intranasal challenge of 2 ml of an HP PRRSV field strain (Vietnam_PRRSV_AGES/568-30FC/13; GenBank accession number KM588915). Serum samples were taken from piglets of group 1 before, seven and 21 days after vaccination and additionally in all 20 study piglets before and at days three, seven, ten, 14, 21 and 28 after challenge. Housing, animal care and experimental protocol of both trials were approved by the local ethics committee (Agency of the Government in Lower Austria, Department of Agrarian Law).

Additionally, serum samples from 301 pigs aged between seven month and three years from four continuously monitored PRRSV negative boar studs from Germany and from one German and one Austrian pig breeding farm (all belonging to PRRSV category IV according to Holtkamp et al. [23]) were tested (group S). The serum samples of these pigs were obtained during routine monitoring. In the farms from which the samples of group S were derived, the > PRRSV PCR and ELISA routinely performed did not produce any positive results.

### Detection of PRRSV-RNA by real-time RT-PCR

All serum samples of groups V and N were tested for the presence of PRRSV RNA by real-time RT-PCR. The RNA extraction was performed using the Freedom EVO^®^ 150 (Tecan, Grödig, Austria) automated platform, and the Nucleospin^®^ 96 Virus and the Nucleospin® Virus Core kits (Macherey-Nagel, GenXpress, Wiener Neudorf, Austria) following the manufacturers’ instructions. The samples were then analysed with a commercial real-time RT-PCR assay for simultaneous detection and differentiation between PRRSV type 1 and type 2 genotypes (TaqMan^®^ NA and EU PRRSV Reagents and Controls, ThermoFisher Scientific, Vienna, Austria) as described previously [12].

### Detection of PRRSV antibodies by ELISA

All of the serum samples from groups V and N were tested for antibodies against PRRSV by the following indirect ELISAs: a) IDEXX PRRS X3 Ab ELISA – in the following called IDEXX, b) INgezim PRRS 2.0 (Ingenasa, Madrid, Spain) – INgezim, c) Civtest suis PRRS A/S plus (Laboratorios HIPRA, Amer, Spain) – HIPRA A/S, d) Civtest suis PRRS E/S plus (Laboratorios HIPRA) – HIPRA E/S, e) pigtype® PRRSV Ab (QIAGEN, Leipzig, Germany) – QIAGEN and f) PRRSV CHECK ELISA (Analytik Jena, aj Roboscreen, Leipzig, Germany) – AJ. All ELISAs tested are commercially available.

In order to determine the specificity, all 301 serum samples of group S were tested with the HIPRA A/S ELISA, 276 of them with the QIAGEN ELISA and 244 of them with the AJ ELISA. Specificity data for the other tested ELISAs, determined with the same sample panel, were published previously [12].

The IDEXX, INgezim and QIAGEN ELISAs are based on the nucleocapsid as antigen [15]. The cut-off for these ELISA was calculated at a sample/positive (S/P) value of 0.4. The HIPRA ELISAs are coated with a glycoprotein rich extract as antigen which is produced by obtaining whole PRRS virus particles with envelope from a non-cell associated PRRSV culture with subsequent solubilizing of glycoproteins with detergents, according to the manufacturer. The cut-off is determined at 20 % of the positive control OD value. The AJ ELISA is based on a coating with inactivated virus, according to the manufacturer. Samples with OD values > 0.5 measured with the AJ ELISA were defined as positive. To be valid, the OD value of the positive control had to be > 1.0 and the OD value of the negative control must be < 0.25. All ELISAs were performed according to manufacturers’ instructions.

### Statistical analysis

The results of the ELISAs measured in the serum samples of group V and N were described as positive and negative for PRRSV antibodies. Additionally, the median, quartiles and 95 % confidence interval of S/P or OD values for each sampling point were evaluated and pictured in boxplots. The correlation coefficient after Spearman was used to test for correlations between the S/P or OD values of the ELISAs and for correlation of the positive and negative results. The agreement between the ELISAs was calculated with the kappa coefficient (κ) using the results of groups V and N and interpreted according to Landis and Koch [19]. The specificity of the ELISAs was determined using the pre-study samples from group V (before vaccination) and group N (before challenge) and all of the samples from group S.
